# Natural Teeth without Roots

**Published:** 1919-03

**Authors:** Archibald R. Gardner


### NATURAL TEETH WITHOUT ROOTS

BY ARCHIBALD R. GARDNER, D.D.S.

An interesting, and what I believe to be unique case of natural teeth, upon which the roots were so incomplete as to be practically absent, presented in the form of a 16 year old boy of good size and apparently in good health. He was brought to the office by his father who said that the boy's teeth were all coming loose and asked that something be done to preserve them.

An examination showed that fourteen natural teeth, with practically perfect crowns, remained in the mouth. The other teeth had been lost, but without any knowledge by the patient or the parents that they were unusual in any way.

To test the degree of looseness in teeth apparently so perfect, slight pressure was applied to the crowns in different directions. They moved with a freedom which seemed to show that there was no process about them. As the amount of movement was too great to permit their retention in fixed positions, and as it was evident that no process could develop while such extensive and continuous movement existed, it seemed necessary to advise extraction. The first tooth was extracted with such ease as to prompt an immediate examination, which showed that the root was merely rudimentary. This was found to be the case with the other teeth.—*Dental Digest.*

